# Suppress to Forget: The Effect of a Mindfulness-Based Strategy during an Emotional Item-Directed Forgetting Paradigm

**DOI:** 10.3389/fpsyg.2017.00432

**Published:** 2017-03-22

**Authors:** Olga L. Gamboa, Javier Garcia-Campayo, Teresa Müller, Frederic von Wegner

**Affiliations:** ^1^Department of Neurology and Brain Imaging Center, Goethe University, Frankfurt am MainGermany; ^2^Department of Child and Adolescent Psychiatry, Medical Faculty, University of Cologne, CologneGermany; ^3^Red de Investigación en Atención Primaria, BarcelonaSpain; ^4^Miguel Servet Hospital, University of Zaragoza, ZaragozaSpain; ^5^Epilepsy Center Rhein-Main and Brain Imaging Center, Goethe University, Frankfurt am MainGermany

**Keywords:** directed forgetting, inhibition, working memory, attention, mindfulness

## Abstract

Forgetting is a common phenomenon in everyday life. Although it often has negative connotations, forgetting is an important adaptive mechanism to avoid loading the memory storage with irrelevant information. A very important aspect of forgetting is its interaction with emotion. Affective events are often granted special and priority treatment over neutral ones with regards to memory storage. As a consequence, emotional information is more resistant to extinction than neutral information. It has been suggested that intentional forgetting serves as a mechanism to cope with unwanted or disruptive emotional memories and the main goal of this study was to assess forgetting of emotional auditory material using the item-method directed forgetting (DF) paradigm using a forgetting strategy based on mindfulness as a means to enhance DF. Contrary to our prediction, the mindfulness-based strategy not only did not improve DF but reduced it for neutral material. These results suggest that an interaction between processes such as response inhibition and attention is required for intentional forgetting to succeed.

## Introduction

At some point in our lives, we all have wished to know the formula that will allow us to erase from our minds certain events that cause us pain, uneasiness, and discomfort. This desire increases when these events permanently invade our thoughts, thus affecting our wellbeing. Although forgetting is a common phenomenon in everyday life, deliberately forgetting painful or unwanted memories is a difficult task. Commonly known as a memory crash, the benefits of forgetting are not perceived intuitively. In order to successfully carry out our everyday duties it is necessary to ignore irrelevant or outdated information that could interfere with effective task performance. It is in this scenario that forgetting provides us with an important adaptive mechanism to avoid loading the memory storage with inappropriate information (Bjork, 1989). Thus, there are two sides to forgetting: an undesirable one, in which unintentional processes lead to unsuccessful attempts to deliberately maintain and/or recover information (“incidental forgetting”) and a desirable one, which controls memory contents in a way that efficiently processes or recovers only relevant information (“intentional forgetting”) (Wylie et al., 2008; Fawcett and Taylor, 2012).

Among the different experimental paradigms used to study intentional forgetting in the lab setting, we have selected the item-method directed forgetting (DF) paradigm. In this method items are first studied individually, until the delivery of an instruction to either remember (R) or forget (F). After a short break, participants are asked to recall the items independent of the given instruction. Indeed, information under the “forget” instruction is more difficult to recall than information under the “remember” instruction: this effect is called directed forgetting (MacLeod, 1989). Typically, two contrasting hypotheses have been proposed to explain DF. (1) The selective rehearsal/passive decay hypothesis considers the forgetting process as a passive mechanism. According to this hypothesis, after an item is presented, it engages rehearsal until the instruction (R or F) is released. Selective treatment including differential encoding and increased rehearsal is granted to items followed by the R instruction, while items followed by the F instruction are ignored, experiencing passive memory decay (Basden et al., 1993). (2) The attentional inhibition – executive control hypothesis considers the forgetting process as an active mechanism. This hypothesis states that once the F instruction appears, active inhibition of the item to forget is engaged in order to remove it from working memory and to prevent its future activation (Zacks et al., 1996). At the same time, executive control functions actively withdraw processing resources from the items to be forgotten, limiting their attentional resources while consequently boosting the rehearsal of the items to be remembered (Hourihan and Taylor, 2006; Wylie et al., 2008).

Currently there is clear evidence that intentional forgetting is in fact an active cognitive process. (Anderson et al., 2004; Depue et al., 2007; Wylie et al., 2008; Nowicka et al., 2011; Oztekin and Badre, 2011; Benoit and Anderson, 2012).

Research in the field of forgetting has experienced an increase after recognizing this process as a key piece in efficient mnemonic function (Kuhl et al., 2007; Welberg, 2007). Being able to ignore irrelevant or outdated information is essential to balance the cognitive load. People have a predisposition to remembering negative experiences more profoundly than neutral ones as this has been evolutionary crucial as a means of survival. As a consequence we find ourselves trying to forget painful or uncomfortable memories on a day to day basis. Several studies have shown that emotionally arousing stimuli profit from unequivocal memory enhancement making them more difficult to suppress (Buchanan and Lovallo, 2001; Sharot et al., 2004; Anderson et al., 2006; Rimmele et al., 2011; Otani et al., 2012) and therefore, resistant to extinction (Kobayashi and Tanno, 2012). It has also been reported that emotionally negative information raises the demands of cognitive resources in order to achieve forgetfulness (Nowicka et al., 2011; Lee, 2012; Yang et al., 2012) and even when intentional forgetting is achieved, it is transient since negative memories regain strength when inhibitory processes are not present (Bjork, 1989; Norby et al., 2010). Research performed on intentional forgetting paired with emotion in the clinical environment has shown impaired DF (Wilhelm et al., 1996; Korfine and Hooley, 2000; McNallya et al., 2004; Joormann et al., 2009; Jaafari et al., 2011; Wingenfeld et al., 2012). Patients with obsessive–compulsive disorder were less able to forget negative material in comparison to positive or neutral items (Wilhelm et al., 1996). Similarly, patients with depression, anxiety, and somatization disorders, showed a poor DF for negative stimuli when compared with neutral material (Wingenfeld et al., 2012; Yang et al., 2016). In addition when instructed to forget, patients with borderline personality disorder exposed to borderline, neutral and positive words remembered significantly more borderline words than the control group (Korfine and Hooley, 2000).

The resistance of negative or unwanted emotional memories to be erased could find an explanation through the ironic process theory (Norby et al., 2010). This theory claims that voluntary inhibition of thoughts consists of two processes: (1) a consciously active operating mechanism, that searches for any thought different to the unwanted thought and (2) a less demanding unconscious monitoring mechanism, that controls for the right performance of the operating process by keeping an eye on any cue that may evoke the unwanted thought (Wegner, 1994, 2009; Wenzlaff and Wegner, 2000). Under situations of cognitive load and distress, the monitoring mechanism tends to work defectively, increasing the salience of the unwanted thoughts causing what is called “the ironic effect.” It has been proposed that using techniques aimed at releasing the need for control may be useful to moderate the intensity of the ironic effect by means of balancing the cognitive resources between the operative and monitoring systems (Bach and Hayes, 2002; Wegner, 2009). Along these lines, a potentially successful approach could be the use of mindfulness techniques. Mindfulness has been defined as a way of paying attention moment to moment in a receptive manner with non-judgmental acceptance (Kabat-Zinn, 1990; Baer, 2003). Thus, mindfulness is an emotionally non-reactive state in which thoughts, emotions and situations are allowed to be as they are without suppressing them and classifying them as good or bad (Kang et al., 2013; Rosenstreich, 2016)

Research in mindfulness has shown that, among others, mindfulness interventions have a significant positive effect in mood and affective processes (Brown and Ryan, 2003), emotion regulation (Arch and Craske, 2006) and release of negativity bias in thought and memory (Alberts and Thewissen, 2011; Kiken and Shook, 2014). Particularly, focused breathing a technique based on mindfulness of breath instructions has shown to be an effective strategy for emotion regulation (Arch and Craske, 2006) and thought suppression (Ju and Lien, 2016) in individuals naïve to mindfulness practices.

Hoping to gain valuable insight regarding the suppression of unwanted memories, our main goal was to evaluate DF by using a modified item-method DF paradigm, in which participants are instructed to use visual imagery to encode auditory emotional material in combination with a strategy to forget based on mindfulness of breath technique. With this procedure we expected to observe enhanced forgetting in the group using a mindfulness-based strategy (Ju and Lien, 2016). Moreover, due to the non-judgmental nature of the strategy in which pleasant and unpleasant experiences or thoughts are approached in the same way and without distinction (Kabat-Zinn and Hanh, 2009) we did not expect to observe differences associated to the emotional valence in the set of words to be forgotten.

## Materials and Methods

### Subjects

A total of 38 healthy volunteers (23 females, mean age = 26.5, *SD* = 5.49) divided in two groups of 19 (control condition: 7 males, mean age = 26.1, *SD* = 5.25; mindfulness condition: 8 males, mean age = 26.2, *SD* = 5.74) participated in this study. All subjects were native German speakers and right-handed according to the Edinburgh handedness inventory (Oldfield, 1971). All subjects were informed about all aspects of the experiments and written informed consent was obtained from each participant on the day of the experimental session. We conformed to the Declaration of Helsinki and the experimental protocol was approved by the Ethics Committee of the Goethe University, Frankfurt.

### Experimental Design

**FIGURE 1:**
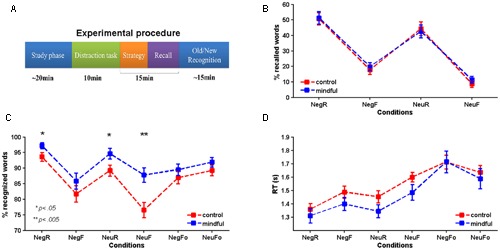
**Task and behavioral results for both groups. (A)** Experimental procedure for the whole experimental session. **(B)** Mean percentage of words recalled as a function of the instruction and emotional valence. **(C)** Mean percentage of words correctly recognized as old [tbr (NegR and NeuR) and tbf (NegF and NeuF) words] or new [Foil (NegFo and NeuFo) words)] as a function of the emotional valence. Significant differences between groups have been indicated with (*p < 0.05 and **p < 0.005). **(D)** Reaction times (RT) of words correctly recognized as old or new. Bars represent standard errors of the mean (SEM). NegR, negative remember; NegF, negative forgetting; NeuR, neutral remember; NeuF, neutral forgetting; NegFo, negative foil; NeuFo, neutral foil.

Participants attended just one experimental session. Short-term and working memory were assessed through the digit span test (digit span forward: mean_control = 8.78, *SD* = 1.70; mean_mindfulness = 8.05, *SD* = 1.39, digit span backward: mean_con = 7.11, *SD* = 1.28; mean_mind = 7.32, *SD* = 1.29). There were no differences between groups (*p* = 0.593). The experimental session consisted of (a) a study phase, (b) 15 min rest (distraction task), (c) a test phase (recalling and recognition tests, **Figure 1A**). Participants in the mindfulness condition were instructed about the focused breathing technique right before the start of the study phase.

### Procedures

#### Stimuli

The 164 words were selected from the **Berlin Affective Word List – Reloaded (BAWL-R)**, a German database with more than 2900 German words containing normative ratings for emotional valence, emotional arousal and imageability (Vo et al., 2009). The sets were composed of 80 negative nouns (negative) and 80 neutral nouns. Negative and neutral words were different as to the emotional valence (*F*_1,162_ = 2659.405, *p* < 0.0001) and emotional arousal (*F*_1,162_ = 5880.464, *p* < 0.0001), but similar in terms of imageability (*F*_1,162_ = 0.385, *p* = 0.536). Additionally, words to be studied were kept similar in length for each condition (*F*_3,76_ = 0.97, *p* = 0.414). Two sets of 80 words equally distributed for category were created (40 items/emotion): one set will be presented during the study phase and the second set will be used as foil items (new words) during recognition test. In the study phase, half of the items of each emotion (20 items) were randomly selected and assigned to either the remember (R) or the forget (F) conditions.

Male and female words using a neutral tone (at 16 kHz linear PCM, with the amplitude normalized at the root-mean-square value) were created by a human voice generator program (AT&T Labs Natural Voices Text-to-Speech Demo and Free Natural Readers^1^).

#### Focused Breathing

Participants in both groups were naïve to mindfulness practices. Before starting the task, participants in the mindfulness condition received instructions about the focused breathing technique and experienced a 10-min mindfulness intervention. Once the participants were seated on a chair, with the feet flat on the floor, eyes closed and body relaxed, they were first instructed to take three deep, slow, and relaxing breaths. This was followed by the instruction to pay attention to their body sensations. Next, they were asked to attend their breath, following its full cycle in their mind’s eye. After some time, they were asked to explore their breath: where it could be felt stronger (nose, throat, chest.), and when this happened (inhalation/exhalation). Subsequently, the session ended by coming back to observing the full cycle of the breath. Taking three final deep, slow and relaxing breaths and opening the eyes whenever they felt ready. After the intervention all participants reported a full understanding of the technique and a sense of relaxation and wellbeing.

#### Directed Forgetting Task

The item-method DF paradigm has two parts, the study and test phases. During the study phase participants were instructed to perform the tasks with their eyes closed and to secure a minimum level of attention participants were required to classify each word as negative or neutral by button press. Auditory stimuli were presented and responses (classification as negative or neutral) were recorded using Presentation software (Neurobehavioral Systems). Before the beginning of the experiment, the sound volume for stimulus presentation was adjusted individually to a level at which participants confirmed they could reliably hear the stimuli. Moreover, participants performed a training session of 10 trials with five negative and five neutral words to guarantee full understanding of the task. Words used during this part were different to the items used during the experimental section and were not considered during the evaluation of the task.

Participants in the mindfulness condition were encouraged to practice the focused breathing technique during this trial period and not to wait for the real task to start.

##### Study phase (encoding)

Words were delivered individually (1 s). Participants were asked to rapidly evaluate the valence of the word (negative or neutral) by pressing a button and instructed to think about a meaningful image related to the word (3.5 s). After that, either the “forget” or “remember” instruction was given (0.5 s), the post – cue length varied randomly from trial to trial and lasted ~ 6 – 7 s. The order of each trial was randomized with the constraint of no more than three consecutive trials of the same kind (same instruction and valence).

Participants in the control group were encouraged to create their own strategies in order to memorize the to-be-remembered words (tbr) and to discard the to-be-forgotten (tbf) words. They were informed that the tbf words were distractors (unwanted items) and it was important to try to keep them away from interfering with the memory task. They were also told that these distractors would not be included in the test phase.

Participants in the mindfulness group were also encouraged to create their own remembering strategy but to switch to focused breathing each time the instruction to forget was delivered. They were instructed not to resist whatever happened during the forgetting period, avoiding judgment (allowing the moment, thoughts and emotions to be as they are) and always trying to gently return to the breath. Special emphasis was given to the fact that this technique should only be used during the forgetting phase. They were also informed that the tbf words were distractors that will try to interfere with the memory task which would not be included in the test phase.

Once the Study phase ended, the participants of both groups performed an unrelated distraction task (that consisted of finding the differences in two apparently identical pictures) for 10 min. During the last 5 min of the resting session, subjects were asked about their remembering and forgetting strategies. To avoid primacy and recency effects (Capitani et al., 1992), four additional words, (two pairs of negative – neutral words) were introduced at the beginning and end of the study phase; these words were excluded from the analyses.

##### Test phase (recalling – recognition)

During this part the participants executed two tasks:

(a) Recalling task: participants were asked to write down all the words they were able to remember from the study phase, regardless of the given instruction.
(b) Recognition task: tbr and tbf words were presented intermixed pseudorandomly with an equal number of foils. Using two buttons, participants categorized the word either as “old” if recognized from the study phase (regardless of the instruction) or as “new” if not recognized.

### Analyses

#### Strategy

The forgetting strategies were classified according to themes concerning the cognitive processes related to the description given by the participants in both the control group during the forgetting period and the response to the strategy in the mindfulness group.

#### Behavioral Data Analyses

A 3-ways mixed ANOVA (2 × 2 × 2) with between–subject factor Group (control – mindful), and within-subjects factors Emotion (negative – neutral) and Instruction (Remember – Forget) was used to investigate the differences in the recalling task.

Similarly, for the recognition task a 3-ways mixed ANOVA (2 × 2 × 3) with between–subject factor Group (control – mindful), and within-subjects factors Emotion (negative – neutral) and Type (Remember – Forget – Foil) was used to assess changes in accuracy (correctly recognized words as old or new) and their reaction times.

Sphericity was assumed according to Mauchly’s test, and Greenhouse-Geisser correction was used when sphericity was violated. Statistical tests were performed using SPSS version 20.0. (SPSS Inc, Chicago, IL, USA. *p*-values ≤ 0.05 were considered statistically significant.

## Results

### Behavioral Data

#### Strategy

There were common themes in the strategies for the control group and for the response to the mindfulness strategy in the mindfulness group. In the control group: two strategies were used: 100% of the participants used a shift in attention (18/19 attended tbr words and 1/19 other), while 78.94% of the participants performed an inhibitory response after delivery of the F instruction (15/19). The mindfulness condition responded in two different ways to the breathing strategy: 78.94% of the participants had intrusive thoughts (15/19 of tbr words, 11/19 of tbf words and 1/19 other), while 42.10% of the participants performed suppression after delivery of the F instruction (8/19). In several cases the two observed strategies were jointly present in the same individual (see Supplementary Table S1).

#### Recall Task

Recalling rates (see **Figure 1B** and **Table 1**) did not differ between groups (*F*_1,36_ = 0.076, *p* = 0.784, $\eta_{P}^{2}$ = 0.002). However, significant differences were obtained for the main factor Emotion (*F*_1,36_ = 17.402, *p* < 0.001, $\eta_{P}^{2}$ = 0.326), with participants recalling more negative than neutral words (*p* < 0.001). The factor Instruction also showed significant differences (*F*_1,36_ = 170.879, *p* < 0.001, $\eta_{P}^{2}$ = 0.826), tbr words were better recalled than tbf words (*p* < 0.001). Finally, none of the interactions among the factors were significant (*F*_1,36_ ≤ 0.316, *p* ≥ 0.578, $\eta_{P}^{2}$≤ 0.009).

**Table 1:** Summary behavioral data.

Emotion	Type	% Recall words (Mean ± SE)		% Recognized words (Mean ± SE)		RT (s) (Mean ± SE)	
		Control	Mindful	Control	Mindful	Control	Mindful
Negative	R	50.79 ± 3.92	51.38 ± 3.98	93.67 ± 1.39	97.24 ± 0.99*	1.36 ± 0.04	1.31 ± 0.05
	F	17.88 ± 3.11	19.90 ± 2.68	81.72 ± 2.65	85.86 ± 2.61	1.49 ± 0.05	1.39 ± 0.05
	Fo			86.96 ± 1.68	89.56 ± 1.82	1.72 ± 0.05	1.72 ± 0.08
Neutral	R	44.31 ± 4.41	42.67 ± 4.27	89.27 ± 1.79	94.64 ± 1.72*	1.45 ± 0.05	1.34 ± 0.05
	F	8.68 ± 2.12	11.43 ± 2.14	76.56 ± 2.50	87.78 ± 2.34*	1.60 ± 0.03	1.48 ± 0.06
	Fo			89.26 ± 1.73	91.93 ± 1.51	1.64 ± 0.05	1.59 ± 0.08

Mean percentage and standard error of recalled and recognized words, reaction times (RT) in seconds for the recognition task are also reported. *p < 0.05. R, remember; F, forget; Fo, foil.

#### Recognition Task

Mean and standard errors for accuracy and reaction times of the task are summarized in **Table 1**.

(a) Accuracy: A 2 (Group) × 2 (Emotion) × 3 (Type) mixed design ANOVA conducted on the recognition scores (**Figure 1C**) showed a significant effect for the between-subjects factor Group. Surprisingly, the mindfulness group recognized significantly more words than the control group (*F*_1,36_ = 9.199, *p* = 0.004, $\eta_{P}^{2}$ = 0.204). Further, *post hoc* pairwise comparison showed that the mindfulness group significantly recognized more neutral tbf (*p* = 0.003) and tbr (negative, *p* = 0.045; and neutral, *p* = 0.039) words than the control group. No differences were observed regarding the Foil words.

The main factor Type also showed a significant effect (*F*_1.33,47.972_ = 25.095, *p* < 0.001, $\eta_{P}^{2}$ = 0.411). As expected, tbf words were significantly forgotten when compared with tbr (*p* < 0.001) and Foil (*p* = 0.020). While tbr words were better recognized than Foil (*p* = 0.007).

Differences were not significant for the main factor Emotion (*F*_1,36_ = 0.996, *p* = 0.325, $\eta_{P}^{2}$ = 0.027).

However, there was a significant interaction between Emotion and Type (*F*_2,72_ = 4.803, *p* = 0.011, $\eta_{P}^{2}$ = 0.118). Negative tbr words were better recognized than neutral ones (*p* = 0.004). On the contrary in the Foil set, neutral words were more accurately recognized than negative words (*p* = 0.011). In the tbf set, no differences were found (*p* = 0.429).

Negative words in the tbr set were better recognized than in the tbf and Foil (*p*s < 0.001) sets. Differences between accurately recognized negative tbf words and negative Foil words were not significant (*p* = 0.068). In the case of neutral words, higher recognition was obtained for the tbr set when compared to the tbf set. There were no differences between tbr and Foil (*p* = 0.471) but decreased recognition was obtained for neutral tbf words in comparison with neutral Foil words (*p* < 0.001).

(b) Reaction time: A similar 2 × 2 × 3 ANOVA design as the previously described showed significant effects regarding RT (**Figure 1D**) for the main factor Type (*F*_1.584,57.036_ = 52.314, *p* < 0.001, $\eta_{P}^{2}$ = 0.592). Reaction times were significantly shorter for tbr words than for tbf (*p* < 0.001) and Foil (*p* = 0.007) words. Similarly reaction times for tbf words were shorter than Foil words (*p* < 0.001). Neither the main factor Emotion (*F*_1,36_ = 1.952, *p* = 0.171, $\eta_{P}^{2}$ = 0.051) nor the between-subjects factor Group (*F*_1,36_ = 1.100, *p* = 0.301, $\eta_{P}^{2}$ = 0.030) showed significant differences.

A statistically significant interaction between Emotion and Type was also observed regarding RT (*F*_2,72_ = 16.941, *p* < 0.001, $\eta_{P}^{2}$ = 0.320). Negative words were recognized faster than neutral words in both the tbr (*p* = 0.027) and tbf (*p* = 0.001) sets. Yet, reaction times were shorter for neutral Foil words were recognized quicker than negative Foil words (*p* < 0.001).

Negative and neutral words in the tbr set were recognized faster than in the tbf and Foil (*p*s < 0.001) sets. Reaction times for negative tbf words were shorter than for negative Foil words (*p* < 0.001), while reaction times for neutral words in both sets were not statistically significant (*p* = 0.064).

## Discussion

The aim of our study was to enhance DF by using a mindfulness-based strategy. Contrary to our expectations the mindfulness group did not show improved forgetting but even showed a significant enhancement in recognition rates for neutral tbf material.

These results are interesting because of two reasons: (1) they support the hypothesis of forgetting as an active mechanism and (2) challenge the view of the ironic process theory which states that resistance to unwanted thoughts increases their salience. They also reinforce the idea that forgetting is a complex process that requires a tactful interplay between active response inhibition and attention mechanisms for it to be successful. This is in agreement with the attentional inhibition - executive control hypothesis which suggests that unwanted information (in this case the tbf items) is actively suppressed to prevent its access to memory and to limit its attentional resources (Zacks and Hasher, 1994; Zacks et al., 1996), facilitating in this way tbr rehearsal (Hourihan and Taylor, 2006; Wylie et al., 2008).

The idea behind the use of a mindfulness-based strategy was to facilitate the process of forgetting by releasing the tension due to the resistance to forget and aiding the reallocation of attentional resources away from the unwanted items. Previous research in mindfulness has shown that after a short mindfulness of breath (focused breathing) session participants without previous mindfulness training experienced less negative thoughts (Kiken and Shook, 2014) and remembered less negative words in a memory task (Alberts and Thewissen, 2011). However, we did not observe facilitation in forgetting negative words. On the contrary we obtained increased recognition of neutral tbf items and enhanced recognition of the tbr items (regardless of their emotional valence). A possible explanation of these results can be found through the recently proposed non-monotonic plasticity hypothesis by Detre et al. (2013). They propose that memory survival depends on a U-shaped interaction between memory activation and memory strengthening/weakening. Successful memory suppression will be possible if competition for attentional resources exists between a moderately activated unwanted item and a strongly activated desirable item (Detre et al., 2013; Lewis-Peacock and Norman, 2014; Fawcett et al., 2016). Thus, under competing conditions, moderately activated (unwanted) items have a higher probability to be forgotten compared to items with lower and higher activations (Detre et al., 2013). Knowing that emotionally charged stimuli have an unequivocal memory enhancement (Buchanan and Lovallo, 2001; Sharot et al., 2004; Anderson et al., 2006; Otani et al., 2012) it is likely that memory representations of negative tbf items were active enough (moderate level) to trigger suppression. In other words, negative tbf items entered the competition for attentional resources against highly active tbr items (according to participant’s reports) causing their own suppression. Meanwhile, neutral items having insufficient salience (low level of activation) failed to elicit suppression, increasing their chances to secure a place in memory (Detre et al., 2013; Fawcett et al., 2016).

Furthermore, tbr words in the mindfulness group experienced enhanced recognition when compared to the control group. Research has shown that mindfulness affects attention mechanism by increasing their efficiency (Jha et al., 2007; Semple, 2010) and improved ability to focus has been observed after one session of a brief mindfulness intervention (Diaz, 2013), which could be the reason for the observed results.

Another finding was the lack of effect observed due to the mindfulness-based intervention during the recall task. The difference between the recalling and recognition results may be due to the different mechanisms underlying these two processes. The process of recall involves response production and response identification, whereas during the process of recognition only the identification phase is required (Tulving and Watkins, 1973; Watkins and Gardiner, 1979). Moreover, partial learning of the items presented during the study phase could be enough for a correct performance during the recognition task but not enough to ensure a successful recall task (Tversky, 1973).

In line with previous studies, aside from the strategy, we found that for both the recalling and recognition tasks, negative material was better remembered and more resistant to being removed from memory and that items instructed to forget were worse recognized than those instructed to remember (Nowicka et al., 2011; Bailey and Chapman, 2012; Yang et al., 2012). A close inspection of the forgetting strategies used by the participants shows that the interaction of attentional control mechanisms with suppression is key in the DF process. Relocation of attentional resources favoring rehearsal of the tbr items was the preferred strategy of the control group (94% of the participants) to support intentional forgetting, while around 78% of the participants made use of some type of suppression to disregard the tbf words. For the mindfulness group, however, the current task proved to be more challenging. Despite being instructed to “simply” be aware of the breathing after the forgetting instruction, participants experienced intrusion of the tbr (73%) and/or tbf items (57%) and used some type of suppression (42%) to avoid keeping the tbf words in memory.

**FIGURE 2:**
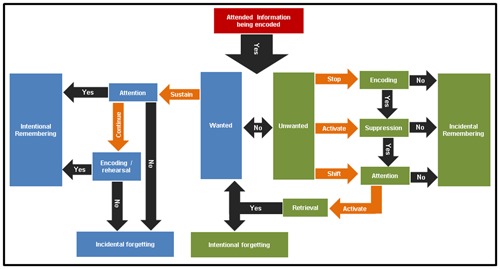
**Forgetting process.** Schema based on the strategies reported by the participants during the forgetting instruction. It shows two loops: on the right side (blue) the “intentional remembering loop” that includes “incidental forgetting”; on the left side (green) the “intentional forgetting loop” which also includes “incidental remembering.” Forgetting being a very complex process seems to make use of both loops in order to be successful.

Based on the forgetting strategies of the control group, the complex mechanism observed at the behavioral level could be summarized as follows: after a word is presented in our experiment, an evaluation should be made and a meaningful image must be created and held in memory until the instruction to either forget or remember is given. When the remember instruction is delivered, word encoding is strengthened by intensive rehearsal, most likely securing a place in memory and leading to intentional remembering. If, on the contrary, representations of wanted information are weakly attended or poorly encoded they may end up being incidentally forgotten (Remembering loop, left side-blue **Figure 2**). However, if the instruction delivered is forget, at least three steps are required to occur: (1) the word-image must be dropped to stop encoding, (2) at the same time the representation of the item should be suppressed to avoid retrieval and (3) the item should be drastically unattended (shift in attention) to avoid further encoding. The attentional shift reported by our participants typically occurred toward wanted items, thus requiring their retrieval and therefore activating the intentional remembering loop. If all the previous conditions are met it is possible that intentional forgetting occurs, otherwise the unwanted item will result in being incidentally remembered (Forgetting loop, right side-green, **Figure 2**). This is coherent with the definition of cognitive inhibition as described by Harnishfeger (1995) (Harnishfeger, 1995; Aron, 2007). According to the reports given by the participants, the same strategy used to forget negative items was used for neutral items. Therefore in this initial scheme no differences have been addressed regarding emotional valence. It will be interesting to further explore this point in future studies, in which items with positive valence are also included to have a bigger picture of the influence of emotional valence during forgetting processes.

One of the limitations of the study was to perform the task only with participants naïve to mindfulness practices. Although mindfulness is a tool that can be used at any given time, without having years of training, according to the reports of the mindfulness group the focused breathing task was challenging. In future work, we plan to explore DF mechanisms with expert mindfulness meditators using neuroimaging techniques, this will allow us to better evaluate the relationship between a more efficient attention mechanism (Jha et al., 2007) and response inhibition.

We found that DF does not improve by using a mindfulness-based strategy; on the contrary, we observed a significant enhancement in recognition rates for neutral tbf material. Our results are in line with the notion that selective attentional inhibition mediates the DF effect (Zacks et al., 1996; Wylie et al., 2008; Nowicka et al., 2011; Bastin et al., 2012) and that such a mechanism is critical for the regulation of working memory content and therefore for the correct function of the cognitive system (Zacks and Hasher, 1994). Additionally, a close interaction between wanted and unwanted information seems to be needed to enable effective DF (Detre et al., 2013; Lewis-Peacock and Norman, 2014; Fawcett et al., 2016) and contrary to common beliefs, we observed that a certain level of resistance to the presence of the unwanted memory is necessary if wishing to attain forgetfulness. The mechanism through which we successfully forget unwanted information is extremely complex and easily prone to failure. Therefore it is important to further investigate the DF mechanisms and the ways in which it can be improved as a first step toward unraveling new therapeutic applications. The progress of intentional forgetting research has relevant implications at the clinical level, mainly in pathologies associated with troubling, intrusive thoughts. Understanding these mechanisms could allow the development of coping strategies that may lead to effective suppression of unwanted memories to keep these disorders under control.

## Author Contributions

OG: Conception and design of the work, data collection, data analysis and interpretation, writing manuscript, critical revision of the article, and final approval of the version to be published. JG-C: Conception and design of the work, data interpretation, critical revision of the article, and final approval of the version to be published. TM: Data collection, data analysis, and final approval of the version to be published. FvW: Conception and design of the work, data analysis, critical revision of the article, and final approval of the version to be published.

## Conflict of Interest Statement

The authors declare that the research was conducted in the absence of any commercial or financial relationships that could be construed as a potential conflict of interest.

## Footnotes

1 http://www.naturalreaders.com/index.html
