# Supplementary material for: Suppress to Forget: The Effect of a Mindfulness-Based Strategy during an Emotional Item-Directed Forgetting Paradigm
Source: Front Psychol. 2017 Mar 22;8:432. doi: 10.3389/fpsyg.2017.00432 (PMC5360695; doi:10.3389/fpsyg.2017.00432)
Supplement: Supplementary file 1 [file Table_1.DOCX]

Supplementary Material

The Art of Forgetting: Suppress to Forget

**Olga Lucía Gamboa*, Javier Garcia-Campayo, Teresa Müller, Frederic von Wegner**

*** Correspondence:** Dr. Olga Lucía Gamboa. Email: [olgaluciagamboa@](mailto:olgaluciagamboa@)gmail.com

Supplementary Data

# Supplementary Table

| **Control group** | | | | **Mindfulness group** | | | |
| --- | --- | --- | --- | --- | --- | --- | --- |
| **Sub** | **Strategy to forget** | **Shift attention** | **Response inhibition** | **Response to the mindful breathing technique** | **Intrusion** | | **Suppression** |
|  |  | **tbr (√) / others (√)** |  |  | **tbr (√) / others (√)** | **tbf** |  |
| **S1** | Pushing the F word away and started to study the story (last words) | **√** | **√** | The experience with the breathing strategy was helpful and got used to it. Started with remembering the tbr last word since it came to mind. | √ |  |  |
| **S2** | Doing nothing special to push the tbf word away, but recalled the tbr words. | **√** |  | It was difficult in the beginning then got used to it. Kept focusing on the breathing and, sometimes before the instruction came, held the breath. |  |  |  |
| **S3** | Seeing the tbf image in a cloud which then exploded. Then imagined random words no related to the study - mainly neutral words. | **√** | **√** | Was not easy to do the mindful breathing. Switched to the last word he needed to remember and then he focused on the breathing. He thinks the strategy works, but selective remembering can be more effective. Resisting to the words he should remember during the breathing. | √ |  | **√** |
| **S4** | Crossing the image of the tbf word and not to put it into the picture created for the tbr words. Then continue observing the picture with the tbr images. | **√** | **√** | Some words were so strong that it was difficult to focus on the breathing, but then it was possible by imagining herself breathing. |  | **√** |  |
| **S5** | Repeating the tbr words (and images). Strategies were automatic. | **√** |  | Sometimes it was harder to forget because some words fit perfectly in the story created for the tbr words. Some other times the forgetting instruction was a relief because some words were very difficult. The tbr words interfere with the breathing strategy so it was not possible to do the breathing technique every time. Focus was on the tbr words and not on the tbf words. The breathing technique was easier with tbf neutral words. | √ | **√** |  |
| **S6** | The tbf words were automatically pushed away. Then trying to think about the different stories created for the tbr words. | **√** | **√** | Tried to start inhaling a bit deeper than before, after the F instruction the period always started with an inhalation. There were a lot of words. The strategy worked but mainly for neutral words. Half or 1/3 of the time the tbr words came after F instruction. The tbr words came to mind when being more focused on the breathing technique. | √ | **√** |  |
| **S7** | Repeating the tbr words (mostly words, although some images came along). Some tbf words were still remembered because they were related to personal current experiences. | **√** |  | Difficult to execute the task of breathing. Tried to focus on the breath and then the image of the tbr word came and he allowed it to be there. | √ | **√** |  |
| **S8** | Once a word was heard it was added to the story but if the instruction was to forget the word was discarded from the story. Then During the tbr words were repeated. | **√** | **√** | Focus on the belly, imagining it tried to follow the breath. The tbf word kept appearing but for a short period then it was possible to continue with the breathing technique. |  | **√** |  |
| **S9** | Not thinking about the tbf image and word anymore and then repeating the last tbr word. | **√** | **√** | Sometimes having the image and receiving the F instruction, it was easy focusing on the breath except two times. The two times the words fitted in the story for the tbr words. Depending on the words it could be a bit more challenging, so there was need to push away the tbf words. Every three words the remembering story came to mind. The remembering story was more intrusive than the tbf words. | √ | **√** | **√** |
| **S10** | After creating the image if the F instruction came not putting the tbf word inside the story created for the tbr words. Then imagining the tbr images and talking to himself about it. | **√** | **√** | It was like in a drain, falling down. The tbf words came later during the task. No interference from any of the words. During the breathing, the focus was on the breathing. |  | **√** | **√** |
| **S11** | Focusing on the tbr words. | **√** |  | It was possible to focus on the breathing and sometimes on the previous tbr word. Sometimes after the F instruction, there was a relief as it was possible to concentrate on the tbr words. It was a good technique to concentrate on the tbr words. | √ |  |  |
| **S12** | Repeated the tbr words. Focusing on the last tbr words and then the tbf word went away. | **√** | **√** | Breathe the word in, put the tbf word inside the chest and reminding herself to forget the word when breathing out. Then focused on the story while breathing more mindfully. | √ |  | **√** |
| **S13** | Not putting them in the tbr story. Then repeating the tbr words. | **√** | **√** | There was not interference from the tbr words. Some few interference from the noise outside. | √ |  |  |
| **S14** | Actively, trying to hard to focus on the images of tbr words, and actively trying to delete the image of the tbf word. | **√** | **√** | It was exhausting. After the instruction and a breath and the picture of the tbf word went away. During the forgetting period, the tbr words came back. | √ |  | **√** |
| **S15** | Some tbf words were more difficult to discard. He actively tried to forget telling himself to forget. Then trying to continue studying the tbr words. | **√** | **√** | Tried to focus on the breathing on the throat. Ther was a feeling of rushing. The mind went to the tbr words. | √ |  |  |
| **S16** | Not repeating the tbf words in the room created for the tbr words/images, but repeating the tbr images/words as he imagined himself in the room without including the tbf words. | **√** | **√** | After the F instruction there was a breath and recalling of the last tbr word. Sometimes the image of the tbf word was overwhelming. The last tbr word in combination with the breathing helped to relax. The tbf words were pushed away actively. | √ | **√** | **√** |
| **S17** | Did not put the tbf words in the story created for the tbr words. Putting the tbf image away and going back to the tbr story repeating the last words/scenes. Sometimes the negative tbf words came back, in that case tried to actively ignore them and focusing strongly on the scenes of the story. Some words were very difficult because they were similar to others so it was difficult to distinguish which one was where. | **√** | **√** | Tried to push away the tbf words, in the beginning it seemed to work very well, but with the time it was more difficult to put the tbf word away because the brain was insisting in putting it inside the story for the tbr words. So there were both words in mind, the tbr and the tbf words that wanted to fit in the story. | √ | **√** | **√** |
| **S18** | Throwing the tbf words behind him, Not creating a relationship with the tbf words. Not putting them inside the picture built for tbr words. Trying actively not to think about but it was difficult. Some were easy because they did not fit the story. Then repeating the tbr story. | **√** | **√** | Sometimes there was a feeling of being connected with the technique, and took a deep breath. However, sometimes it was not possible to focus on the breath after the F instruction because he could not fully disconnect from the previous remembering task. Sometimes the tbf words interfered. | √ | **√** |  |
| **S20** | Recalling and rehearsing the tbr words Instead of trying to actively forget. If the tbf word was too strong it was difficult to let go, then actively trying to focus on the story created for the tbr words to get distracted from the word. | **√** | **√** | It worked sometimes but if a word fit perfectly into the story for the tbr words it was hard to forget (it was not related to emotional valence). Tried to forget imagining the tbf word was not there. Tried to actively suppress the word. During the breathing there was a relief and then rehearsed or reflect about the remembering story. | √ | **√** | **√** |
|  | **Total** | **19** | **15** | **Total** | **15** | **11** | **8** |

**Table S1. Strategy data.** Subjects’ report on their strategies used during forgetting (control group), and response to the assigned strategy in the mindfulness group. Sub: subjects, tbr: to be remembered words; tbf: to be forgotten words.
